# Supplementary material for: Evolutionary Trend Analysis of Research on 5-ALA Delivery and Theranostic Applications Based on a Scientometrics Study
Source: Pharmaceutics. 2022 Jul 15;14(7):1477. doi: 10.3390/pharmaceutics14071477 (PMC9320574; doi:10.3390/pharmaceutics14071477)
Supplement: Supplementary file 1 [file pharmaceutics-14-01477-s001.zip › pharmaceutics-1726912-supplementary.pdf]

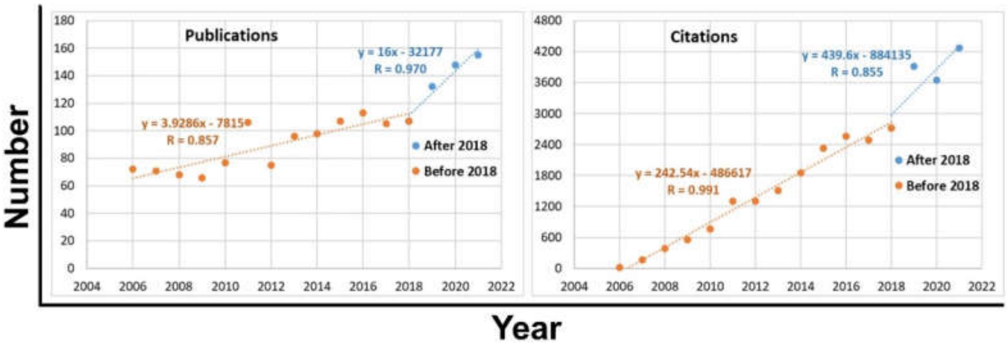

Supplementary Figure S1. The linear regression model before and after 2018 in publication/citation terms. Data were processed using EXCEL 2016.

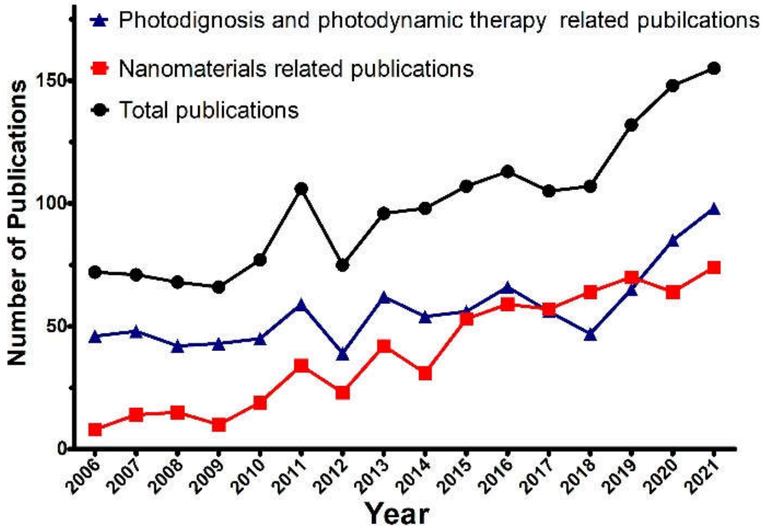

Supplementary Figure S2. The publications comparison between nanomaterials related publications and photodiagnosis/photodynamic therapy related publications. Total publications were retrieved with the formula of TI = (*aminolevulinic acid OR 5 ALA); the nanomaterials related publications were collected in the retrieve formula of TS = (nano*) AND TI = (*aminolevulinic acid OR 5 ALA) or TS = (Protoporphyrin ix OR PpIX. the photodiagnosis/photodynamic therapy related publications were achieved in the retrieve formula of TI = (*aminolevulinic acid OR 5 ALA) AND TS = (photodiagnosis OR photodynamic). All publications were articles only.

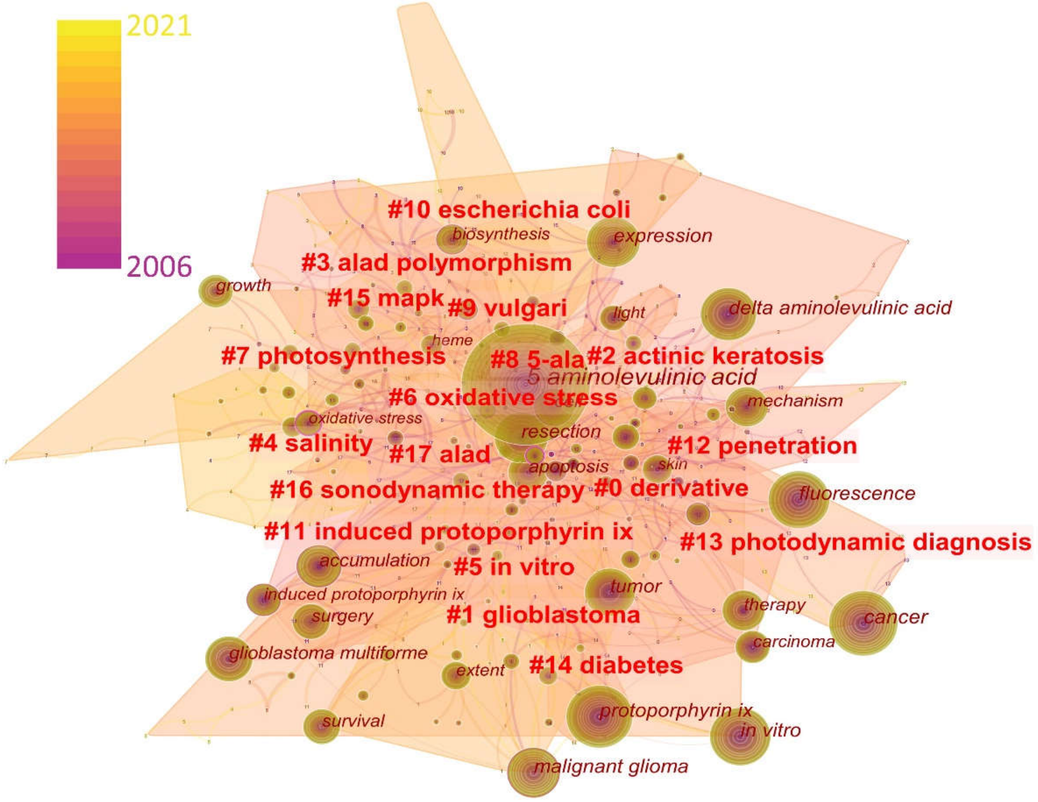

Supplementary Figure S3. The second level cluster analysis of keywords after shielding the common keywords associated with photodiagnosis and photodynamic therapy.

Supplementary Table S1. The top 10 most active funding agencies in 5-ALA research.

Funding Agencies	Publisher's Country	Publication Counts	% of 1595	ACI	*h*-index
National Natural Science Foundation of China	China	198	12.41	15.11	31
Ministry of Education Culture Sports Science and Technology Japan Mext	Japan	118	7.40	14.03	22
National Institutes of Health USA	US	93	5.83	25.04	29
United States Department of Health Human Services	US	93	5.83	25.04	29
Japan Society for The Promotion of Science	Japan	69	4.33	13.14	17
Grants In Aid for Scientific Research Kakenhi	Japan	47	2.95	12.87	15
Nih National Cancer Institute	US	37	2.32	35.03	20
European Commission	European Union	31	1.94	18.55	14
Fundamental Research Funds for The Central Universities	China	18	1.13	16.17	10
Nih National Institute of Neurological Disorders Stroke	US	18	1.13	44.28	13

Supplementary Table S2. The top 10 authors with high between centrality in 5-ALA research.

Rank	Author	Count	Centrality
1	Shunichiro Ogura	24	0.03
2	Motowo Nakajima	43	0.02
3	Masahiro Ishizuka	26	0.02
4	Yuichiro Hagiya	9	0.02
5	Tohru Tanaka	64	0.01
6	Kiwamu Takahashi	34	0.01
7	HIideo Fukuhara	17	0.01
8	Yasutoshi Murayama	8	0.01
9	Fuminori Abe	7	0.01
10	Takuya Ishii	6	0.01
